# Ethics

**Published:** 1904-03

**Authors:** E. A. Bogue


### ETHICS.

By Dr. E. A. Bogue.

Nero is one of the most widely-known names in history; the most detestable character that ever sat upon a throne and wielded the power of a great nation. We seek in vain for any redeeming traits. When, finally, in a frenzy he caused Rome to be set on fire in many places at once, that he might sate his appetite for horrors in a view of the burning capital, and the people arose and demanded vengeance, Nero in his craven fear turned most naturally to lying, and accused the then few and helpless sect of Christians of having done the nefarious deed. At once there arose a persecution so wide, so cruel, so bloody, that in Rome itself but few of the despised sect remained alive. Nero's life was a life of selfishness unrestrained. Nero was an entirely unethical personage.

Kedney expresses wonderment that the ideal of a human brotherhood, and the adjustment of moral duties thence arising, did not enter into the minds of the Greek philosophers. It seems to us so easy. But in the Greek times the political conditions for the realization of an ideal of human brotherhood did not exist, nor did they think it possible that they could exist. It was in the Roman dominion, with its quasi-universality, that first dawned clearly upon the human mind the possibility of such a brotherhood being realized. Its feasibility has been growing more and more distinct for the imagination ever since.

That out of the bosom of the most exclusive of all peoples, the Jews, should have arisen the One who first gave the conception distinct utterance is, to say the least, a notable event in the history of humankind. It was then that was announced for the first time, in that most marvelous of all discourses ever given, the Sermon on the Mount: "Give, and it shall be given unto you; good measure, pressed down, shaken together, and running over, shall men give into your bosom. For with the same measure that ye mete withal, it shall be measured to you again." Owen Meredith has well put the matter when he says: "We are our own fates; our own deeds are our doomsmen. Man's life was made, not for men's creeds, but men's actions." And almost the same idea is expressed by George Eliot in "Adam Bede:" "Our deeds determine us, as much as we determine our deeds."

I infer that every gentleman before me is a practitioner of my craft. It is scarcely sixty years ago since the Medical College of Baltimore refused to acknowledge that dentistry was in any sense a branch of medicine. It is scarce six months since His Majesty's Privy Council decided to continue the present course of dental teachings in England, lest any change in the present curriculum might result in the severance of dental surgery from general medicine.

In America we have recognized that the best way to learn to do a thing is to do it, and we have often started doing it before our minds were sufficiently stored with the knowledge of the past, or even with the present, or even with the principles that underlie our doing. There are, consequently, some matters connected with the higher portions of our profession that have been more sedulously pursued by strictly medical men than among dentists. Among these is that intangible, but quite perceptible, actuality called ethics—which means morals. The lower down in the scale of humanity the less the knowledge of morals, because there are no morals to have knowledge of; but as civilization advances, ethics, or the knowledge of morals, becomes more widely disseminated, until we reach a strictly Christian community, when the science crystallizes into the aphorism promulgated by the Divine

Leader of men: "As ye would that others should do unto you, do ye even so to them." A Golden Rule indeed, and the epitome of all codes of morals, or codes of ethics, which have ever hitherto been devised.

In all civilized communities there are individuals who are backward, and who have not reached the level of the others. For the backward ones, "codes" of ethics have seemed necessary, and have been adopted, specifying one thing after another with the dictum "Thou Shalt" or "Thou shalt not" attached.

Man started out on his career as a predatory animal going, like Satan, up and down in the world seeking whom he might devour. To-day the highest intelligences have become convinced of the value of Christian teachings, and that the highest ethical culture, the greatest unselfishness, and the utmost kindness of each to the other are the best, and are even the most profitable when considered from the lower standpoint of business morals. The greatest among us are those who are seeking what they can give, and not what they can get.

Francis Bacon, in his preface to the "Elements of the Common Law of England," says: "I hold every man a debtor to his profession, from the which, as men of course do seek to receive countenance and profit, so ought they of duty to endeavor themselves, by way of amends, to be of help and ornament thereunto." How many among us have sedulously pursued this course, and have so striven to direct not only our own individual courses, but the courses of those bodies with which we are professionally connected, as to acquit ourselves of the duty we owe to our profession? And yet a society is but a collection of individuals, and is subject to the same laws as govern individuals.

When working on the constitution and by-laws of the American Dental Club of Paris, the question of adopting the code of ethics of the American Dental Association came up. One of the members of that committee said, "I have a suggestion to make, and that is that we simply put the three words, 'The Golden Rule,' in place of the usual code of ethics." The committee unanimously adopted the suggestion.

I will ask permission to add Hamlet's way of putting it.

This above all: to thine own self be true,
And it must follow, as the night the day,
Thou canst not then be false to any man.

"If you should find in some unfortunate patient's mouth that owing to extraction the teeth are occluded differently from what they once were and certain lower cusps are working upon some of the inclined planes above in such a manner as to endanger the fillings put in by yourself or some other practitioner, or even to endanger the tooth itself, it would be quite ethical to grind down that filling or an occluding surface and so prolong the life of the filling or tooth, or both, just as you would want it done for yourself." To claim that conditions brought into being by lapse of time which endanger the patient's well-being is not your affair to ask over again the question of Cain, "Am I my brother's keeper?" If it be not "doing the things we ought not to have done," it is "leaving undone the things we ought to have done."—November *Cosmos.*

